# Insights on Carbon Neutrality by Photocatalytic Conversion of Small Molecules into Value-Added Chemicals or Fuels

**DOI:** 10.1021/accountsmr.2c00095

**Published:** 2022-11-04

**Authors:** Haimiao Jiao, Chao Wang, Lunqiao Xiong, Junwang Tang

## Abstract

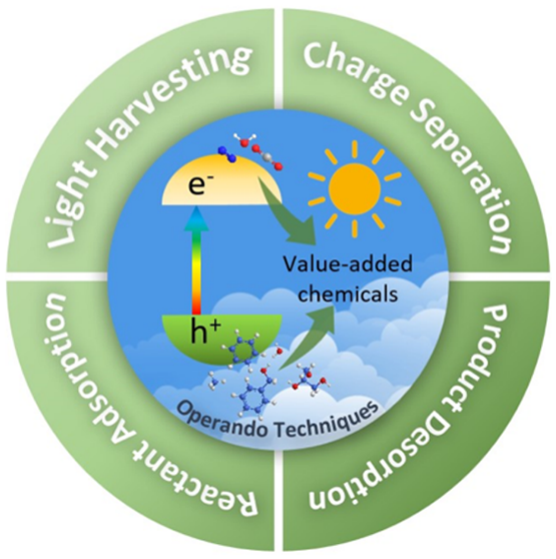

Photocatalytic conversion of small molecules (including H_2_O, CO_2_, N_2_, CH_4_, and benzene) into value-added chemicals or fuels (e.g., H_2_, NH_3_, C_2_^+^, etc.) is a promising strategy to cope with both the worldwide increasing energy demand and greenhouse gas emission in both energy sectors and chemical industry, thus paving an effective way to carbon neutrality. On the other hand, compared with conventionally thermo- or electrocatalytic processes, photoactivation can convert these very stable small molecules by the unexhausted solar energy, so leading to store solar energy in chemical bonds. Thus, it can effectively reduce the reliance on the nonrenewable fossil fuels and avoid the substantial emission of hazardous gases such as CO_2_, NO_*x*_, and so on while producing valued-added chemicals. For example, semiconductors can absorb solar light to split H_2_O into H_2_ and O_2_ or convert CO_2_ to alcohols, which can then be used as zero or neutral carbon energy sources.

Although many efforts have already been made on photocatalytic small molecule activation, the light–energy conversion efficiency is still rather moderate and the yield of aimed value-added chemicals cannot meet the requirement of large-scale application. The core for these artificial photocatalytic processes is to discover a novel photocatalyst with high efficiency, low cost, and excellent durability. Over the past two decades, the Tang group has discovered a few benchmark photocatalysts (such as dual-metal-loaded metal oxides, atomic photocatalysts, carbon-doped TiO_2_, and polymer heterojunctions, etc.) and investigated them for photocatalytic conversion of the above-mentioned five robust molecules into value-added chemicals or liquid fuels. Besides, advanced photocatalytic reaction systems including batch and continuous flow membrane reactors have been studied. More importantly, the underlying reaction mechanism of these processes has been thoroughly analyzed using the state-of-the-art static and time-resolved spectroscopies. In this Account, we present the group's recent research progress in search of efficient photocatalysts for these small molecules’ photoactivation. First, the strategies used in the group with respect to three key factors in photocatalysis, including light harvesting, charge separation, and reactant adsorption/product desorption, are comprehensively analyzed with the aim to provide a clear strategy for efficient photocatalyst design toward small and robust molecule photoactivation under ambient conditions. The application of in situ and operando techniques on charge carrier dynamics and reaction pathway analysis used in the group are next discussed. Finally, we point out the key challenges and future research directions toward each specific small molecule’s photoactivation process.

## 1 Introduction

Facile activation and selective conversion of small molecules such as H_2_O, CO_2_, N_2_, CH_4_, and C_6_H_6_ into value-added chemicals or green fuels is a very promising means for the sustainable development of our society.^1^ Nonetheless, one remaining challenge is to break the inert chemical bonds (e.g., C—H, C=O, and N≡N) therein, which is an endothermic reaction and energy-intensive process. To achieve this, thermocatalysis and electrocatalysis are proposed. For example, the Haber–Bosch process is used for the industrial NH_3_ production, where H_2_ and N_2_ are involved as reactants over iron- or ruthenium-based catalysts under high temperature (*T*: 450 °C) and pressure (*P*: 15 to 30 MPa).^2^ In the electrocatalytic CO_2_ reduction system, a much more negative potential compared with the normal hydrogen electrode (NHE) is needed to overcome the high CO_2_ reaction barrier and produce CO_2_^–^ radicals.^3^ However, the substantial energy input in the form of heat or electricity in these processes cause a huge amount of energy consumption and expensive capital cost. Therefore, an efficient, environmentally friendly, and economic system for small molecule activation and selective transformation is highly desirable.

Inspired by natural photosynthesis where green plants capture solar energy to convert CO_2_ and H_2_O into carbohydrates, the artificial photocatalytic process has attracted extensive attention for small molecule activation by a green pathway.^4^ Solar light is an abundant and renewable energy source that can be captured and utilized to generate green fuels, thus reducing the dependency on the fossil fuels and avoiding some exhausted gases emissions (e.g., CO_2_, NO_*x*_, SO_2_, etc.). In addition, this solar-driven process can effectively promote small molecule activation in the presence of catalysts under very moderate conditions. Last, this mild reaction pathway provides a promising strategy for the large-scale application in the future. For instance, a 100 m^2^ arrayed panel reactor based on Al-SrTiO_3_ photocatalyst was constructed to achieve photocatalytic H_2_ production from water with the maximum solar-to-hydrogen (STH) efficiency of 0.76%, which exhibits a great potential for the small molecule activation.^5^

**Figure 1:**
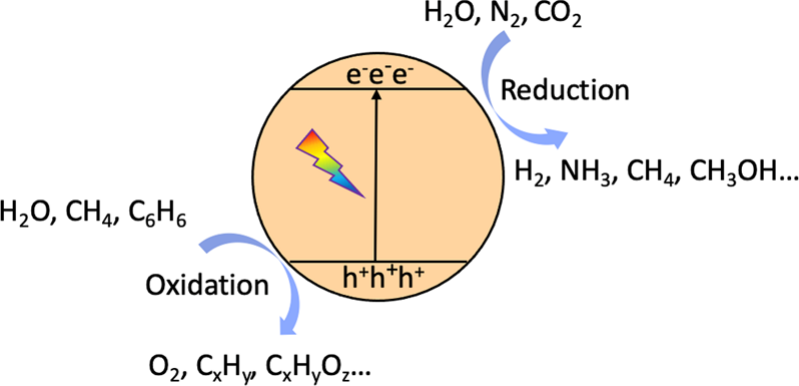
Schematic illustration of photocatalytic conversion of small molecules into value-added chemicals over semiconductors undertaken in our group.

In recent years, various semiconductors such as TiO_2_, g-C_3_N_4_, CdS, ZnO, SrTiO_3_, and so on have been extensively investigated in small molecule photoactivation process.^6−8^ However, it still faces a big challenge about the low conversion efficiency and selectivity due to the activation of these very inert molecules. Thus, it is of paramount importance to develop new photocatalysts to boost the conversion efficiency of small molecules into value-added chemicals or fuels. The reaction mechanism for photoinduced small molecule activation and transformation undertaken in our group is depicted in Figure 1 and has been systematically reviewed by our other work.^2,3,7,9,10^

**Figure 2:**
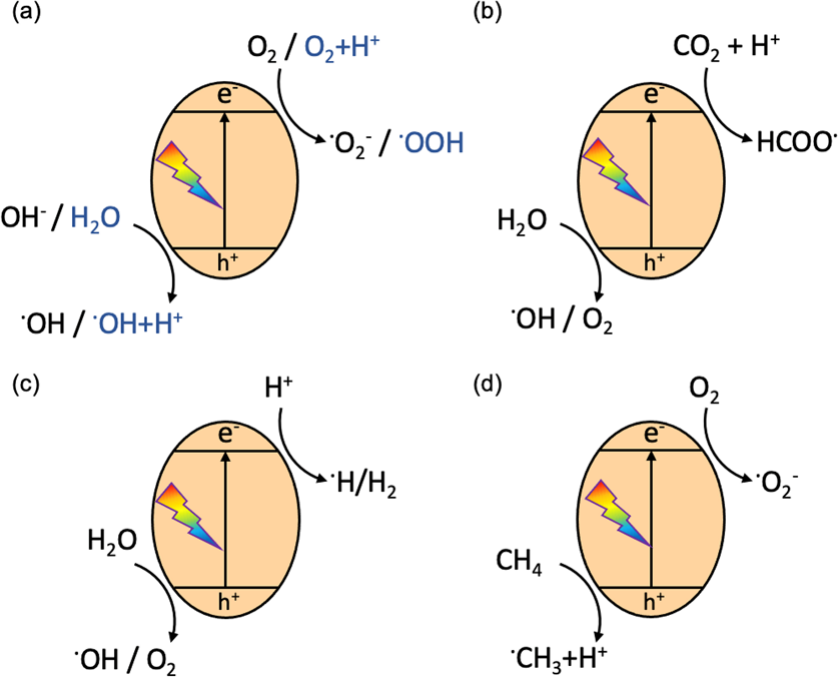
Typical radicals including (a) ^•^O_2_^–^ and ^•^OH, (b) HCOO^•^, (c) ^•^H, and (d) ^•^CH_3_ generated in photocatalytic reactions.

In brief, upon light irradiation, semiconductors are excited by the incident photons with the energy (*hv*) equal to or larger than their bandgap energies (*E*_g_) to generate photoexcited electrons and holes. Next, photogenerated electrons at the conduction band (CB) transfer to electron acceptors to participate in the reduction reactions including H_2_ production, CO_2_ reduction and N_2_ activation, while photogenerated holes at the valence band (VB) migrate to hole acceptors to proceed oxidation reactions involving water, CH_4_, benzene, and alcohols. Nevertheless, some photoexcited electrons and holes directly recombine inside semiconductors or on the semiconductor surface during the migration process, thereby leading to substantial energy loss, which is believed to be the major reason for low solar to fuel conversion efficiency. Therefore, it is very important to explore an effective way to improve the photocatalytic efficiency and inhibit the recombination of photoinduced charge carriers. These aforementioned photocatalytic reactions usually involve complex radical reactions. The reactive oxygen species (ROS) play a significant role in various photocatalytic oxidation reactions when oxygen and water are used in the reaction system. For instance, ^•^O_2_^–^ and ^•^OH are formed from oxygen reduction by electrons, while ^•^OH could be generated from water oxidation by photoinduced holes (Figure 2a). Then, the ROS participates in a series of oxidation reactions to convert the reactants into oxidation products. In photocatalytic CO_2_ reduction, the direct reduction of CO_2_ by photoelectrons to form a ^•^CO_2_^–^ radical is very difficult and requires a reduction potential of −1.9 V vs SHE. Therefore, the first step of CO_2_ reduction is assisted by one proton, to form a formate radical (Figure 2b). In photocatalytic water splitting, ^•^H radicals are first formed from proton reduction by electrons (Figure 2c). Then the coupling reaction of two ^•^H radicals results in the formation of one H_2_ molecule. The methyl radical is the first and the most important intermediate formed in all methane conversion reactions, which results from methane activation by photogenerated holes, with the production of a proton (Figure 2d).

In this critical review, a comprehensive understanding for rational design strategies of highly effective photocatalysts used in our group will be detailed, which will include bandgap engineering for light harvesting, charge separation enhancement, morphology engineering for reactants adsorption/products desorption, and charge separation, as well as operando fundamental observations. Then, the future research direction toward the bottlenecks of photocatalytic conversion of each small molecule will be discussed with an aim to realize carbon neutrality as soon as possible.

## 2 Strategies to Enhance Photocatalytic Efficiency

In general, the criteria for the choice of photocatalysts which either fit for the reduction and/or oxidation reaction can be summarized as follows. First, semiconductors must have appropriate conduction or valence band potentials to proceed the corresponding reduction or oxidation reactions.^1^ For example, the electrons in the conduction band must have a more negative potential than the reduction potential of H^+^/H_2_ (0 eV vs NHE) to proceed the H_2_ reduction half-reaction. The water oxidation reaction could occur when photoholes exist in the valence band, which has a more positive potential than the oxidation potential of O_2_/H_2_O (+1.23 eV vs NHE). Besides, the intrinsic properties of n- or p-type conductors also need to be considered.^11^ For instance, when a p-type and a n-type semiconductor are in close and intimate contact to form a p–n heterojunction, the band bending on semiconductor is very significant, which can induce the flow of electrons to the conduction band of the n-type semiconductor and direct the flow of holes to the valence band of the p-type semiconductor. This can greatly enhance the charge separation and improve the photocatalytic performance. Furthermore, it is very crucial to choose suitable cocatalyst loading on semiconductors, which can promote the charge separation and control the reaction pathways/product selectivity.^12^ Very recently, the single-atom Cu on TiO_2_ was proved as an efficient cocatalyst to trap electrons and improve charge transfer.^13^ Accordingly, several improvement strategies will be reviewed systematically by us as shown below, aiming at guiding readers to prepare efficient photocatalyst for photocatalytic conversion of small molecules.

### 2.1 Light Harvesting

#### Defects

The defect engineering on semiconductors can be used to effectively change the band positions, construct active sites and enhance light harvesting to improve the photocatalytic performance.^9^ We recently reported that n-type g-C_3_N_4_ with introduced N-defects and C—OH terminal groups could work as an efficient photocathode.^14^ Combination of open circuit photovoltage decay (OCVD), Mott–Schottky (MS) plot and transit absorption spectroscopy (TAS) analysis proved that the existence of longer-lived shallow-trapped electrons at the microsecond time scale on defective g-C_3_N_4_ after light irradiation contributed to its superior photocathodic properties, which was also strongly supported by the H_2_O_2_ treatment experiments. It could be seen that there was a left shift of the C—OH peak in the *C*_1*s*_ XPS spectra and the ratio of the C—OH peak to N=C—N was increased from 0.043 to 0.066 on the H_2_O_2_-treated reference g-C_3_N_4_ film without defects (ref-g-C_3_N_4_), which indicated the successful introduction of N-defects and C—OH end groups. Accordingly, the treated ref-g-C_3_N_4_ also could exhibit attractive photocathodic performance. Taking these advantages, the optimal defective g-C_3_N_4_ exhibited 100 times higher conductivity and 1000 times longer lifetime of shallow-trapped electrons than those on bulk g-C_3_N_4_. Very recently, one holey graphic carbon nitride with rich bridging-nitrogen defects (D-CNNS) was synthesized for boosted photocatalytic hydrogen production.^15^ After introducing bridging-nitrogen defects, the D-CNNS exhibited an enhanced visible light harvesting compared with bulk carbon nitride (BCN) as a result of the improved n → π* optical transition. The theoretical calculations revealed that the bandgap of D-CNNS was greatly decreased to 2.24 eV as compared to that (2.74 eV) on BCN. Further characterizations showed that D-CNNS exhibited an efficient spatial charge separation. As a result, a superior hydrogen yield of 2497.1 μmol g^–1^ h^–1^ was reached on the optimized D-CNNS(0.3), which was around 41 times higher than that of BCN.

#### Doping

**Figure 3:**
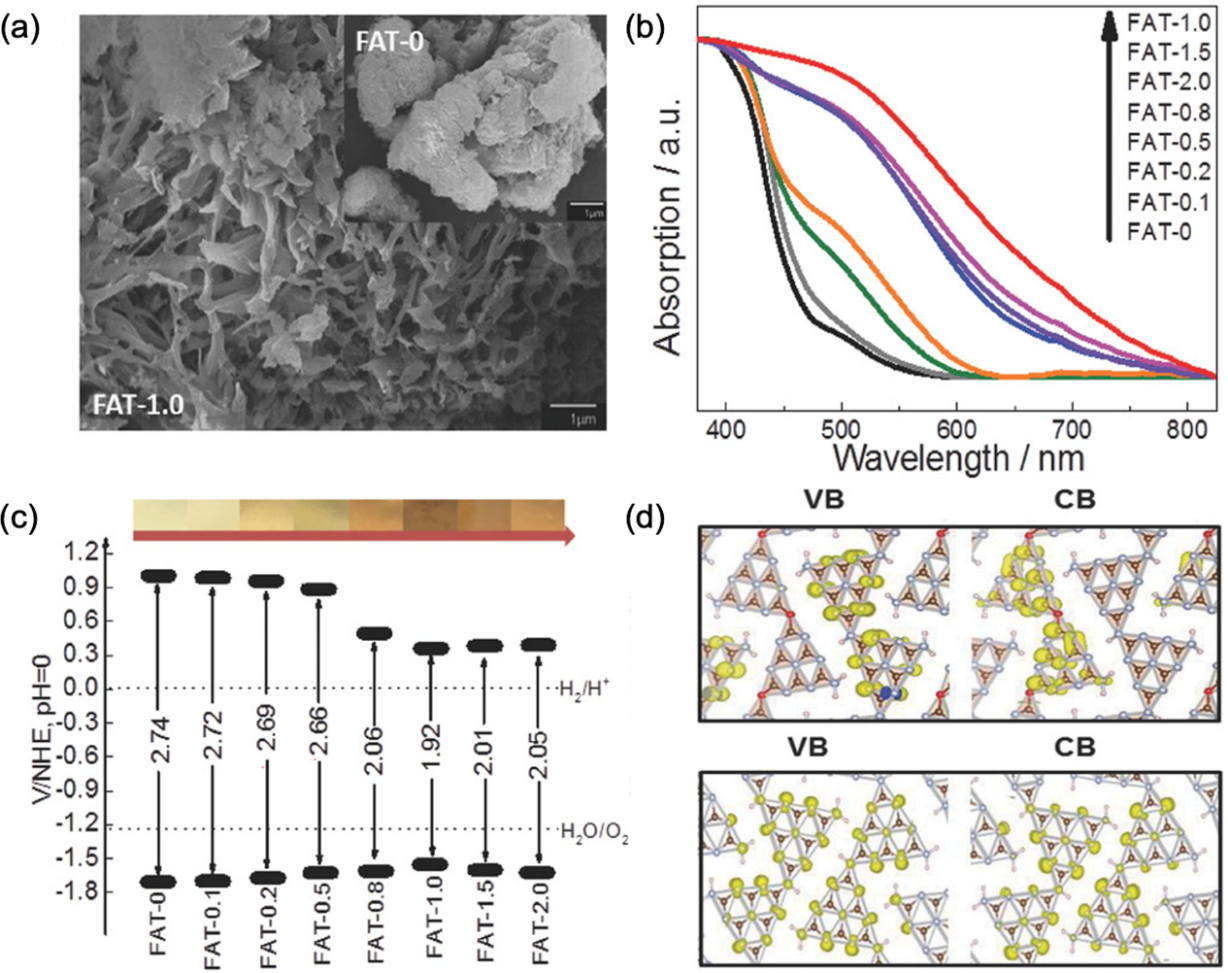
(a) SEM images of formic acid treated polymeric photocatalyst (FAT-1.0). The inset is the pristine carbon nitride (FAT-0). (b) UV–vis DRS spectra, (c) band structures of FATs, and (d) the valence band and conduction band distribution of FAT (upper panel) and pristine carbon nitride (lower panel). Reproduced with permission from ref (18). Copyright 2018 John Wiley and Sons.

The introduction of various dopants into semiconductors is another promising strategy to change their electronic structure and surface property in order to boost their photocatalytic performance.^8,16^ In one of our studies, the controlled hydroxyl/oxygen group was doped in CN_*x*_H_*y*_ during the polymerization process to boost its photocatalytic property for H_2_ evolution.^17^ The DFT results revealed that the heptazine units could be linked by oxygen and nitrogen species, thus leading to a narrow band gap and reduced charge recombination. Besides, the terminal −OH groups on doped CN_*x*_H_*y*_ also increased its surface hydrophilicity, which was beneficial for water adsorption and proton reduction. The optimal hydroxyl-doped carbon nitride with the narrowed band gap of 1.55 eV exhibited an excellent H_2_ production rate of 102 μmol g^–1^ h^–1^, which was 25 times higher than that (4 μmol g^–1^ h^–1^) on pure g-C_3_N_4_ under visible light, together with the apparent quantum efficiency (AQE) of 10.3% and 2.1% at 420 and 500 nm, respectively. Furthermore, we also reported that the well-controlled replacement of nitrogen linkers or terminals in the heptazine structure over CN_*x*_H_*y*_ by formic acid treatment in the abbreviation of FAT-X, where FAT meant formic acid treated polymer and the subsequent X meant the stoichiometric ratio of formic acid to DCDA in the precursors.^18^Figure 3a compared the morphology of oxygen-linked carbon nitride (FAT-1.0) and pristine carbon nitride (FAT-0). The FAT-1.0 exhibited a stacking layered structure rather than a ribbon-like structure of pristine g-C_3_N_4_. Besides, the FAT with different ratio of oxygen linkers exhibited a step-by-step enhanced visible light adsorption (Figure 3b) and the bandgap of the FAT with different amount of oxygen linkers was effectively narrowed (Figure 3c). The DFT calculations proved that the charge efficiency was improved due to the special separation of electrons and holes after the introduction of oxygen-containing linkers (Figure 3d). Therefore, a superior H_2_ evolution rate (HER) of 772 μmol g^–1^ h^–1^ (420 nm < λ > 710 nm) was achieved, which was 18 times higher than that on g-C_3_N_4_, resulting in the benchmark AQY of 2.5% at 500 nm. Incorporating oxygen into the covalent triazine-based frameworks (CTF) was again proved to be an effective strategy in tuning its photocatalytic performance.^19^ Both water reduction and oxidation activities were improved after the modification. In particular, the oxygenated CTF has been remarkably active for oxygen production in a wide operation window from UV to visible, and even to NIR (up to 800 nm), with an unprecedented external quantum efficiency of 1% at 600 nm for water oxidation. Such activity very much matches the solar spectrum.

Besides oxygen doping, modification of carbon nitride with other inorganic elements, such as C and S also plays important roles in manipulation of its photocatalytic activity.^20^ We found that an ultrathin sulfur-doped porous carbon nitride (S—CN) nanosheet was very active for boosting photocatalytic hydrogen production.^21^ Further investigations revealed that the surface area of S—CN was enlarged and the corresponding conduction band was lifted up with the decreasing doping amount of sulfur. Accordingly, an optimal S—CN prepared with 0.1 g of thiocyanuric acid as a precursor showed a remarkable H_2_ yield of 6225.4 μmol g^–1^ h^–1^ (λ > 420 nm), which was around 45 times higher than that on bulk g-C_3_N_4_, with the AQY of 10% at 420 nm.

**Figure 4:**
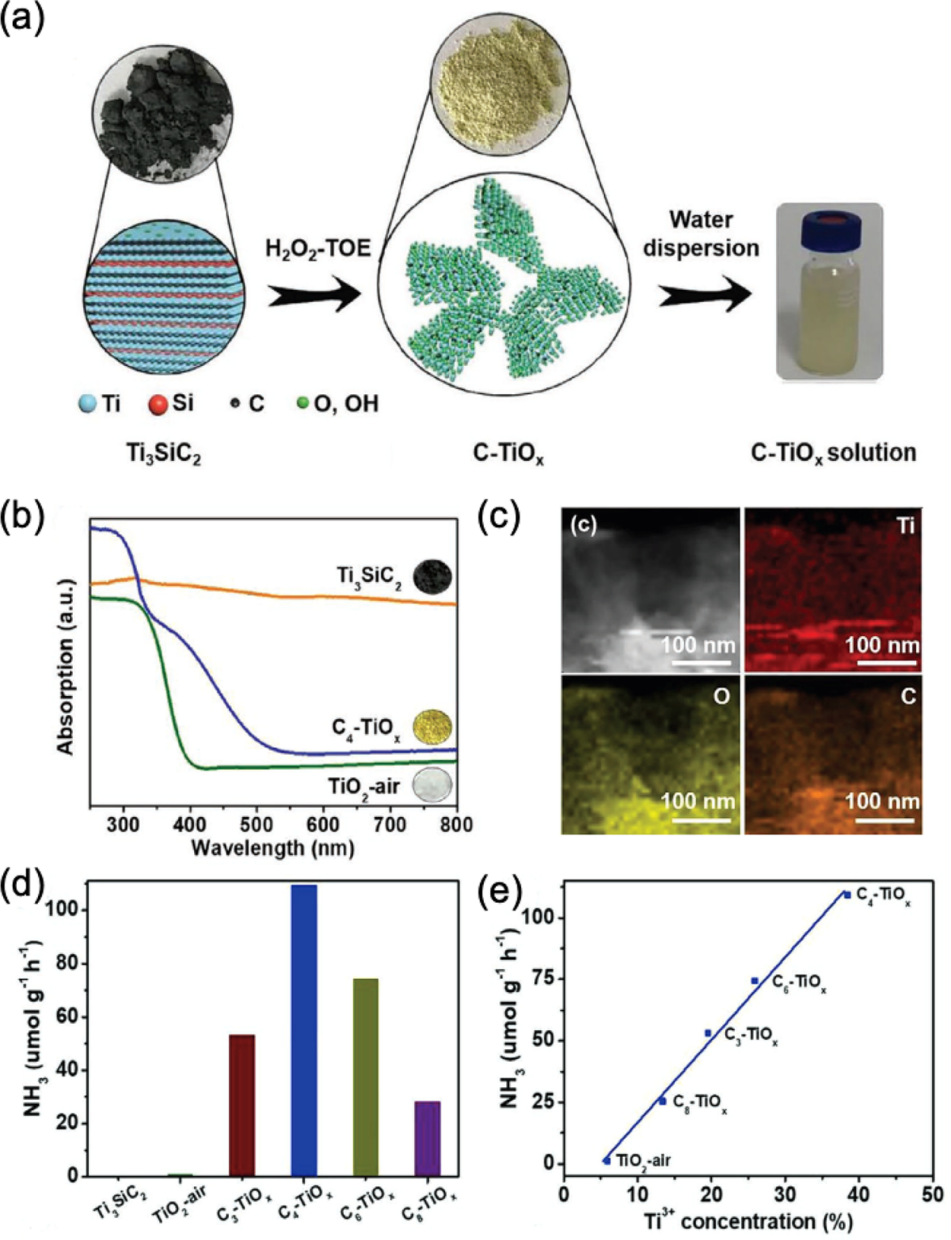
(a) The synthetic route of carbon-doped TiO_2_, (b) UV–vis spectra of the fabricated TiO_2_ photocatalysts, (c) TEM-EDS mapping figures of carbon-doped TiO_2_, (d) the NH_3_ production rate on different catalysts loaded with 5 wt % Ru species as a cocatalyst in the presence of methanol, and (e) the relationship of NH_3_ production rate with Ti^3+^ concentration. Reproduced with permission from ref (23). Copyright 2021 John Wiley and Sons.

In parallel, many reports have mentioned the use of metal-doped TiO_2_ in photocatalytic nitrogen reduction,^22^ compared with it, a few studies focused on using non-metal-component-doped TiO_2_ in this field. Recently, we synthesized carbon-doped TiO_2_ nanosheets with enriched Ti^3+^ by H_2_O_2_-assisted thermal-oxidation etching (TOE) treatment of Ti_3_SiC_2_ MXenes and a subsequent heat treatment (Figure 4a).^23^ The carbon doping over TiO_2_ was proved to effectively induce the formation of Ti^3+^ active sites and dramatically enhance its visible light adsorption (Figure 4b). The elemental mapping spectra of the samples displayed the homogeneous dispersion of C element in the sample, indicating a successful doping of C into the framework of TiO_2_ (Figure 4c). With the synergetic effects of Ru and RuO_2_ for enhanced charge separation, a remarkable NH_3_ yield of 109.3 μmol g^–1^ h^–1^ with the AQE of 1.1% at 400 nm was achieved over 5 wt % Ru/C_4_–TiO_*x*_ under visible light illumination (Figure 4d). Meanwhile, it was discovered that the photocatalytic NH_3_ production performance was in correlation with the concentration of Ti^3+^, rather than the carbon content (Figure 4e). Besides, direct calcination of Ti_3_C_2_ MXenes in air resulted in the formation of carbon-doped TiO_2_ enriched with oxygen vacancies.^24^ The formed oxygen vacancies not only extended the absorption of light but also improved the adsorption of N_2_.

Apart from the aforementioned two strategies, constructing the heterojunction structure is also an effective way to enhance the light harvesting and several works have been reported by our group.^11,25^ For example, the metal-free heterojunction composed of hollow carbon nanospheres (NCS) and graphic carbon nitride (CN) was successfully prepared and used for efficient photocatalytic H_2_ evolution.^26^ Due to the formation of special coupling interface between NCS and CN, the light absorption on CN/NCS was extended to the visible light region, thus leading to improved catalytic performance of CN. Moreover, the formation of the heterojunction structure is also beneficial for the photoinduced charge separation on semiconductors. Thus, more details about the advantages of the heterojunction structure will be discussed in the subsequent charge separation section.

### 2.2 Charge Separation by Cocatalysts Loading and Junction Structure

In this subsection, the major strategies contributing to the improved charge separation including cocatalyst loading and junction structure are emphasized. The other improvement methods with side contribution on charge separation will be briefed in the next sections.

#### Cocatalyst Loading

Cocatalysts loading has been extensively investigated as an efficient strategy to lower the activation energy and equally important to retard the electrons and holes recombination during photocatalysis, thus leading to enhanced photocatalytic activity.^27,28^ Very recently, we reported a ternary Ru/RuO_2_/g-C_3_N_4_ heterostructure for N_2_ photoreduction.^29^ Benefiting from the improved charge separation by loading Ru and RuO_2_ and enhanced N_2_ adsorption and activation on Ru species, the optimal Ru/RuO_2_/g-C_3_N_4_ exhibited an NH_3_ yield of 13.3 μmol g^–1^ h^–1^ under full spectrum irradiation, while almost no NH_3_ were detected on pristine g-C_3_N_4_.

**Figure 5:**
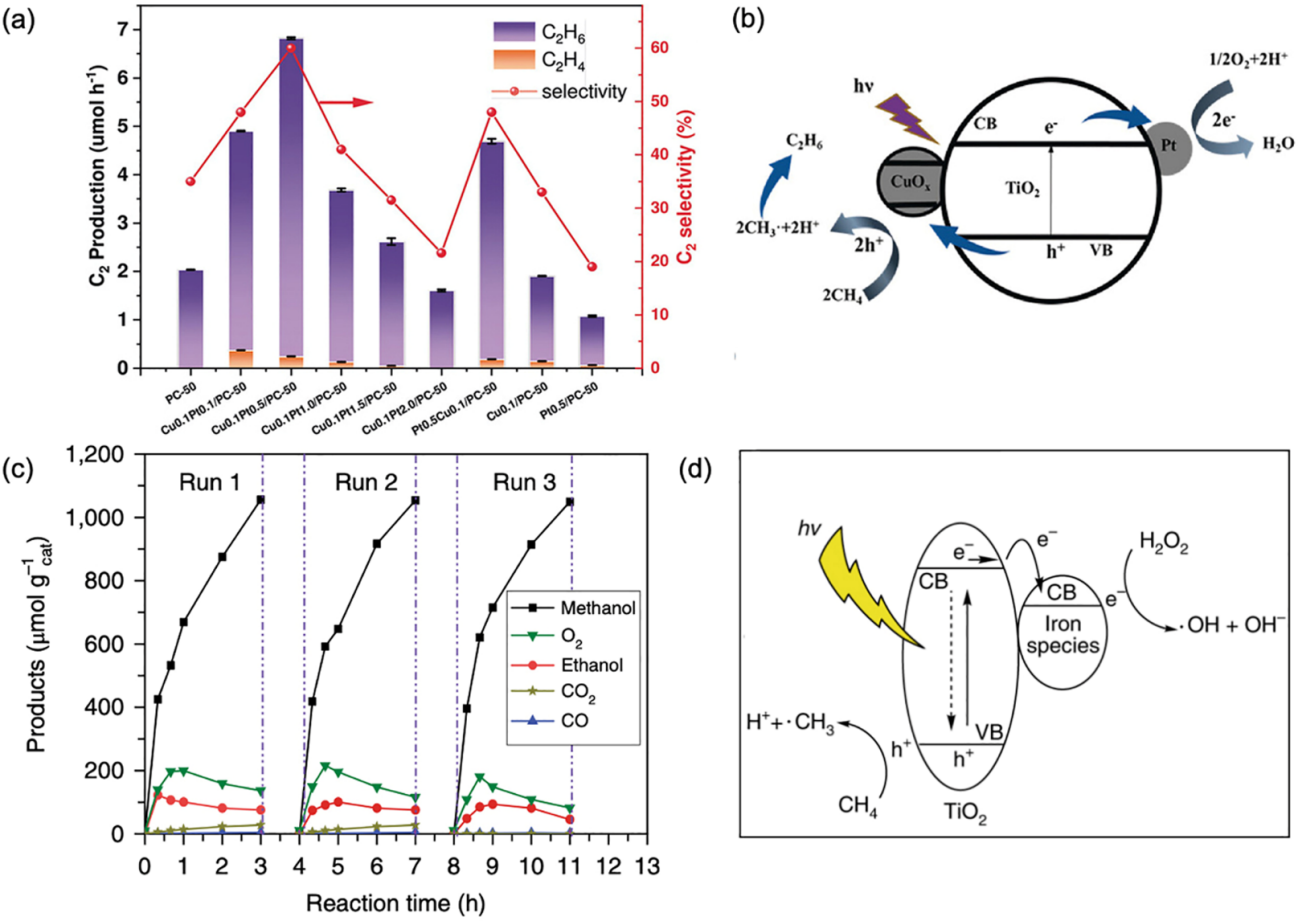
(a) Photocatalytic performance and (b) reaction mechanism of TiO_2_ modified by Pt and CuO_*x*_ cocatalysts for oxidative coupling of methane. Reproduced with permission from ref (30). Copyright 2020 John Wiley and Sons. (c) Product formation rates and (d) photocatalytic reaction pathway of partial oxidation of methane over FeO_*x*_ decorated TiO_2_. Reproduced with permission from ref (31). Copyright 2018 Springer Nature.

In one of our recent studies, Pt and CuO_*x*_ were coloaded on TiO_2_ and used in a flow system for photocatalytic oxidative coupling of methane (Figure 5a,b).^30^ Pt, a widely used electron-acceptor, and CuO_*x*_, which was assumed to be a hole acceptor, ensured maximum separation of photocarriers generated by TiO_2_. Photoholes on CuO_*x*_ with lowered oxidation capability activated methane to generate methyl radicals and protons, avoiding overoxidation. Meanwhile, O_2_ was reduced by electrons on the surface of Pt to produce the O_2_^–^ radical, which cleaned the surface of TiO_2_ from the accumulation of H^+^. As a result, an unprecedentedly high C_2_H_6_ production rate of 68 μmol g^–1^ h^–1^ was achieved.

However, the scarcity and high price of noble metal-based cocatalysts in part limit the large-scale application. Iron species were then decorated on TiO_2_ as a low cost cocatalyst for selective (90% selectivity) methanol production from methane activation with the assistance of H_2_O_2_ (Figure 5c).^31^ There were two functions of the iron species (Figure 5d). First, electrons generated from TiO_2_ transferred toward FeO_*x*_, where decomposition of H_2_O_2_ was accelerated, forming oxidative ^•^OH radicals. In parallel, methane could be activated either by the photoholes or ^•^OH radicals and then by reacting with ^•^OH to generate methanol.

In parallel, a covalent nickel bis-aminothiophenol catalyst as a new noble metal-free cocatalyst was successfully grafted on 2D carbon nitrides (C_3_N_*x*_H_*y*_) for efficient charge separation and photocatalytic H_2_ production.^32^ Time-resolved spectroscopy indicated that the rate of submicrosecond electron transfer in Ni(abt)_2_ covalently bonded photocatalysts was 6 orders of magnitude higher than that (>2 s) in the nongrafted photocatalysts. Besides, the half-lifetime of photoelectrons (7 ms) in the Ni(abt)_2_ grafted C_3_N_*x*_H_*y*_ was 700 times longer than that (10 μs) in the pristine C_3_N_*x*_H_*y*_. Therefore, a remarkable performance on CN_urea_—Ni(abt)_2_ was achieved with the H_2_ yield and turnover frequency of 92 μmol g^–1^ h^–1^ and 9.2 h^–1^ respectively, which were comparable with those (155 μmol g^–1^ h^–1^ and 6.9 h^–1^) on Pt-loaded CN_urea_ under the same experimental conditions. Besides, CN_urea_—Ni(abt)_2_ also exhibited superior durability over 192 h, which was 3 times longer than that on most reported molecular catalyst-based carbon nitrides.

#### Junction Construction

Compared to the photocatalytic system using only one semiconductor, constructing a heterojunction system with two or more photocatalysts such as Z-scheme or type II structure can effectively promote spatial charge separation and enhance the photocatalytic activity.^10,33^ In one of our studies, one metal-free nanojunction (s-BCN) made of boron-doped carbon nitride and bulk carbon nitride was synthesized successfully as a highly efficient photoanode for water oxidation.^34^ Boron-doped carbon nitride could shift the valence band of bulk g-C_3_N_4_ (G-CN) upward from +1.42 V to +1.35 V vs Ag/AgCl (pH 6.5). The driving force between two different valence bands in the junction structure promoted the hole transfer from G-CN to the surface to participate in the following photooxidation reaction. Besides, s-BCN exhibited a 3-fold higher photocurrent intensity than that on G-CN. As a result, the optimal s-BCN (4%) showed an excellent photocurrent density of 103.2 μA cm^–2^ at 1.23 V vs RHE under one sun illumination and a remarkable high incident photon-to-electron conversion efficiency (IPEC) of 10% at 400 nm, which was 10 times higher than that on bulk carbon nitride.

In parallel, to reduce CO_2_ into useful chemicals or fuels, heterostructures based on Cu_2_O were studied as photocatalysts.^35^ For instance, the reduced graphene oxide (RGO)/Cu_2_O heterojunction was synthesized in one step via a microwave-assisted chemical method.^35^ Not only was a 6 times higher CO formation rate compared with bare Cu_2_O obtained, but also the catalyst stability was significantly prolonged. The Cu leaching was as low as 96 ppm in RGO/Cu_2_O, compared to the high leaching of 2670 ppm in Cu_2_O after a 3 h reaction time. Most recently, a cascade Z-scheme system of (001)TiO_2_–g-C_3_N_4_/BiVO_4_ (T-CN/BVNS) was successfully constructed to enhance CO_2_ photocatalytic reduction.^36^ The DFT results revealed that the introduced TiO_2_ as the electron-energy platform could effectively promote the charge transfer and prolong the lifetime of charge carriers. Accordingly, as compared to BVNS, the optimal 5T-15CN/BVNS exhibited around 19 times better performance for CO_2_ photoreduction to CO without any cocatalysts and sacrificial agents under visible light irradiation.

### 2.3 Reactant Adsorption or Charge Separation by Morphology Engineering

#### Facets

To date, effort has been made on the facets control over semiconductors because of their distinct impacts on the band edge location, charge separation, and important reactants’ adsorption.^37^ The photocatalytic oxidation of water using Ag_3_PO_4_ was studied by theoretical modeling and experimental analysis.^38^ First, it was predicted that the {111} facet of Ag_3_PO_4_ displayed the higher surface energy of 1.65 J/m^2^ compared with energies of the {110} and {100} planes. Therefore, a series of Ag_3_PO_4_ nanocrystals with exposed {111}, {100}, and {110} were synthesized. The tetrahedral Ag_3_PO_4_, mainly composed of the {111} facet, showed an oxygen evolution rate of 6 mmol g^–1^ h^–1^, which was 10 times higher than that of cubic Ag_3_PO_4_ with the exposed {100} facet or rhombic dodecahedron Ag_3_PO_4_ with the exposed {110} facet. Furthermore, an internal quantum yield of almost unity for water oxidation was realized at 420 nm, which was the highest performance achieved in photocatalytic water oxidation by visible light. We also found that the selectivity of photocatalytic CO_2_ reduction could be controlled by facet engineering of Cu_2_O nanocrystals.^39^ The octahedral Cu_2_O with the exposed {111} facet was proved to be beneficial for hydrogen evolution, while the Cu_2_O particles consisting of cuboid aggregates could selectively reduce CO_2_ to produce CO. A similar effect was also observed using KTaO_3_.^40^ The charge separation process could be boosted by the facet control on a heterojunction photocatalyst.^41^ It was proved experimentally that the {010} facet of BiVO_4_ and the {002} facet of ZnO were the electron-rich facets, while the {110} plane of BiVO_4_ and the {110} facet of ZnO were the hole-rich planes. Therefore, the delicate growth of ZnO nanorods on BiVO_4_ nanocrystals formed a Z-scheme heterojunction, which produced O_2_ at a rate of 1.36 mmol g^–1^ h^–1^ under visible light irradiation.

#### Nanostructure

**Figure 6:**
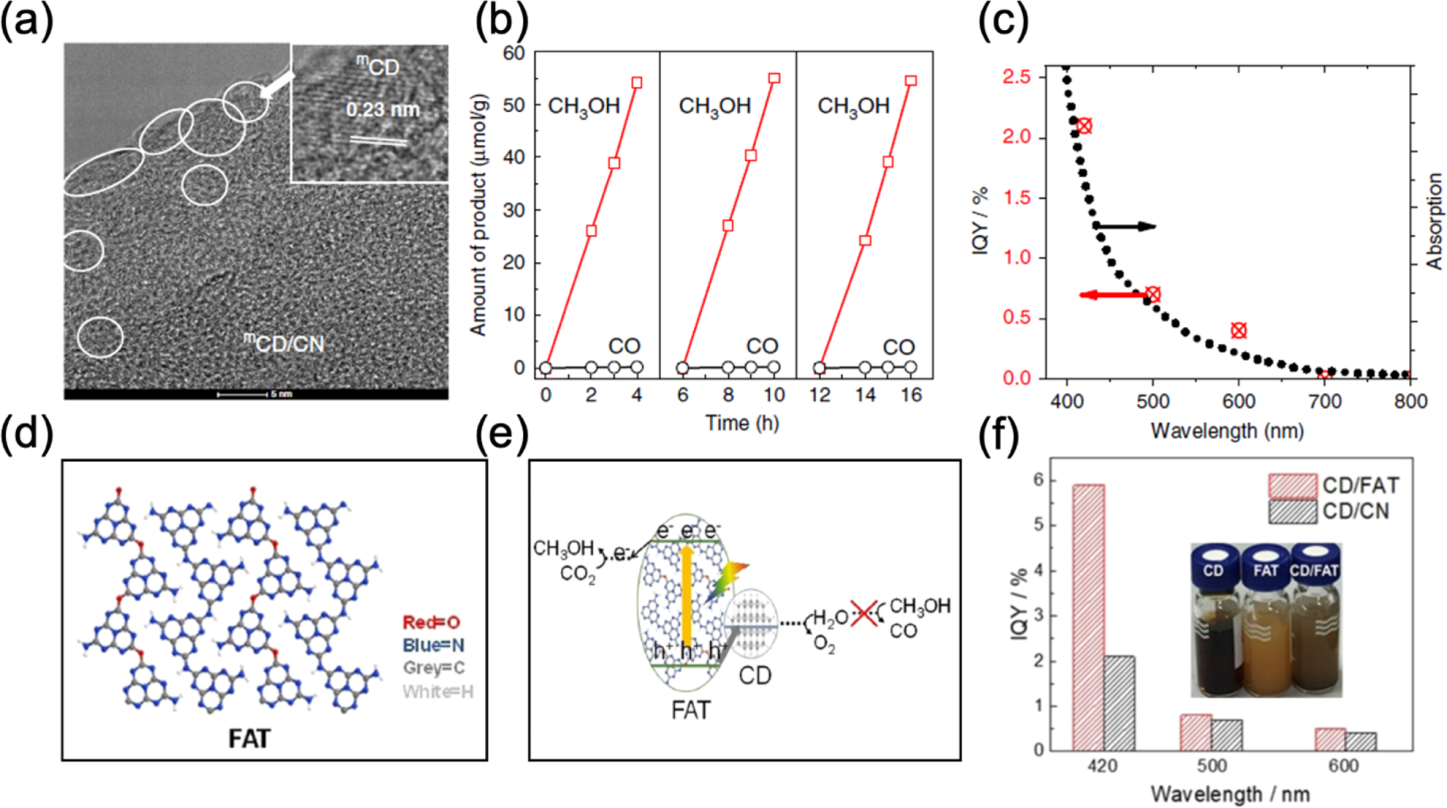
(a) TEM images, (b) methanol formation rate from CO_2_ reduction, and (c) wavelength dependent IQY of ^m^CD modified g-C_3_N_4_. Reproduced with permission from ref (42). Copyright 2020 Springer Nature. (d) Structures of FAT polymer, (e) reaction mechanism of FAT decorated CD for photocatalytic CO_2_ reduction, and (f) the quantum efficiency of CO_2_ photoreduction to methanol by CD decorated g-C_3_N_4_ and FAT photocatalysts. Reproduced with permission from ref (43). Copyright 2021 John Wiley and Sons.

Carbon dots, due to their attractive electrical, optical, and chemical properties, are drawing increasing attention in photocatalysis. The properties of carbon dots can be easily adjusted by tuning their size, composition, and preparation method. Recently, we reported the special hole-accepting carbon dots prepared by the microwave method (^m^CD), which was combined with graphitic carbon nitride (g-C_3_N_4_) to drive photocatalytic carbon dioxide reduction to methanol (Figure 6a–c).^42^ The decoration of ^m^CD on g-C_3_N_4_ promoted the charge separation, water adsorption and methanol desorption processes in photocatalytic CO_2_ reduction. Therefore, a 6-fold enhancement in methanol production rate with nearly 100% selectivity was achieved over the ^m^CD/CN photocatalyst. Combining ^m^CD with another polymeric photocatalyst, FAT (oxygen-doped CN), which displayed a lower methanol oxidation capability, further promoted the CO_2_ reduction process, resulting in a quantum efficiency of 6% at 420 nm (Figure 6d–f).^43^

#### Reactant Adsorption

**Figure 7:**
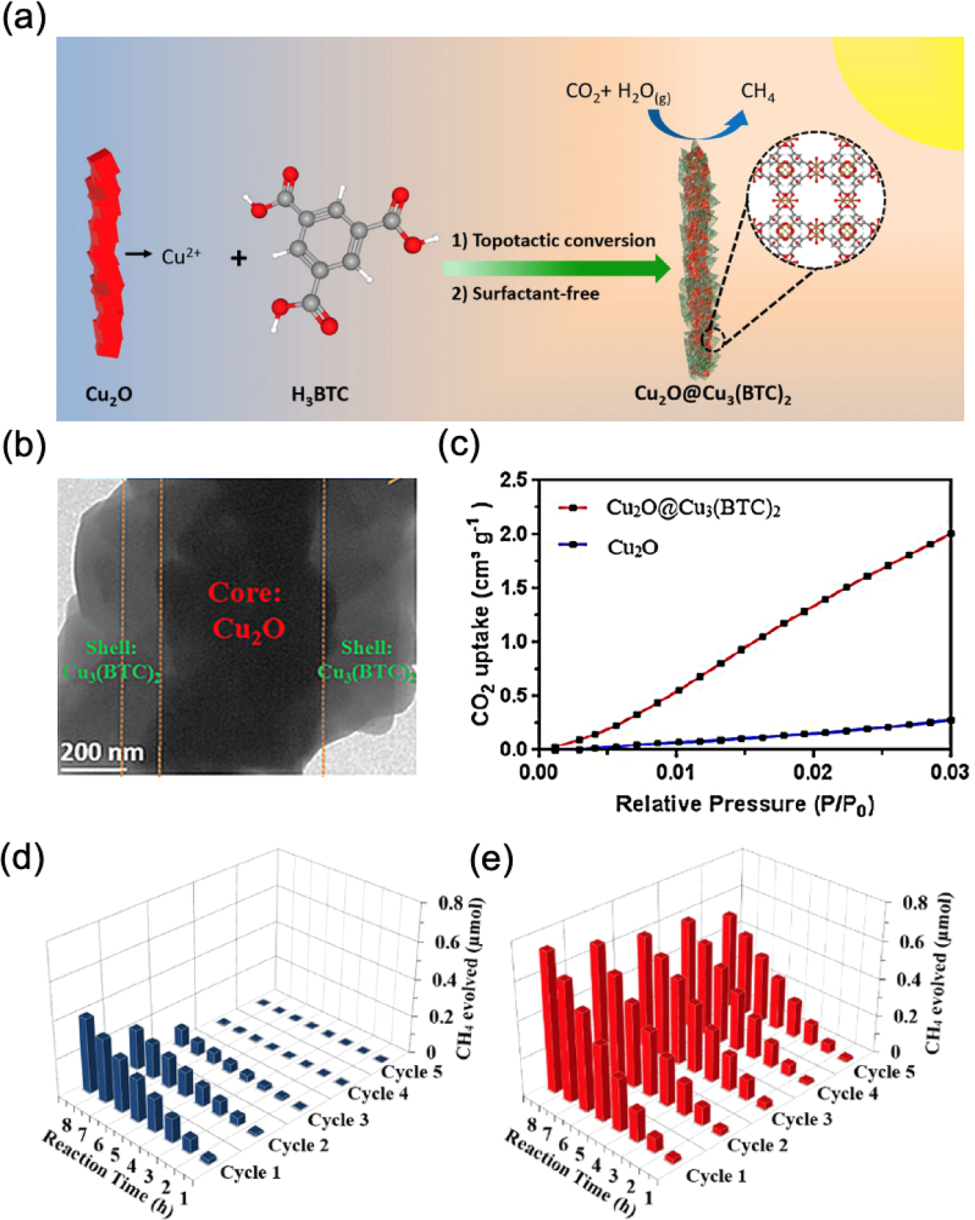
(a) The synthetic process and (b) TEM image of the Cu_2_O@Cu_3_(BTC)_2_ core–shell structure, (c) CO_2_ uptake of Cu_2_O and Cu_2_O@Cu_3_(BTC)_2_, and the photocatalytic CO_2_ reduction for CH_4_ formation performance of (d) Cu_2_O and (e) Cu_2_O@Cu_3_(BTC)_2_. Reproduced with permission from ref (44). Copyright 2021 John Wiley and Sons.

Loading a reactant adsorbent on the photocatalyst is an effective method to achieve the improved adsorption of reactant molecules. A Cu_2_O@Cu_3_(BTC)_2_ core–shell structure was fabricated for the photocatalytic CO_2_ reduction (Figure 7a,b).^44^ Cu_3_(BTC)_2_ on the surface of Cu_2_O could significantly improve the adsorption of CO_2_ due to its special porous structure (Figure 7c). Therefore, abundant CO_2_ molecules were confined near the surface of Cu_2_O photocatalyst. As a result, a ca. 2 time improvement on the photocatalytic CO_2_ reduction into CH_4_ was observed (Figure 7d,e). More importantly, the stability of photocatalyst was improved after loading the Cu_3_(BTC)_2_ metal organic framework onto Cu_2_O (Figure 6e). Recently, we also found that the Ti_3_C_2_ MXenes could act as a N_2_ adsorbent on the surface of TiO_2_ and improve the photocatalytic N_2_ reduction activity.^45^ The DFT calculations suggested that N_2_ has a much higher adsorption energy on MXenes (2.7 eV) than on TiO_2_ (0.2 eV). With the assistance of CH_3_OH as an electron donor, an NH_3_ production rate of 44 μmol g^–1^ h^–1^ was achieved on the optimized 6% Ti_3_C_2_ MXenes-P25 under full spectrum light illumination, which was almost 4 times higher than that of pure P25 TiO_2_.

Structure engineering of semiconductor photocatalysts can also improve the absorbance of reactants. In one of our work, among three types of carbon nitride synthesized from urea, dicyandiamide, and thiourea, urea-derived carbon nitride with the lowest protonation showed the highest photocatalytic hydrogen evolution performance.^46^ Protonation at the linkers or terminals of the photocatalysts resulted in the decreased interaction with the protons, which was the source of produced H_2_. Combined with the high surface area, an extraordinary hydrogen evolution rate of 20000 μmol g^–1^ h^–1^ was obtained over urea-derived carbon nitride. Surface engineering also can alter the intrinsic properties of the catalyst surface and vary its adsorption capabilities. Surface-fluorinate TiO_2_ (F-TiO_2_) showed a special photoinduced hydrophilicity,^47^ as the adsorption of H_2_O molecules on F-TiO_2_ was improved compared with that on bare TiO_2_. As a result, photocatalytic hydrogen evolution from water, aldehyde decomposition and methylene bule degradation were all improved. In particular, the photocatalytic hydrogen evolution rate was improved by more than 2 times over F-TiO_2_ compared with that on bare TiO_2_.

### 2.4 Fundamental Understanding by in Situ and Operando Studies

Time/spatial-resolved operando techniques are powerful tools to monitor the charge carrier dynamics and reaction pathway during the photocatalytic process, thus guiding the improvement of the photocatalytic performance.^48^ In 2014, we employed transient absorption spectroscopy (TAS) to monitor the behavior of charge carriers on Cu_2_O/Ru_*x*_O for CO_2_ photoreduction.^49^ The TAS results revealed that the junction of Cu_2_O/Ru_*x*_O could produce a 2 times higher yield of long-lived (>100 μs) photoexcited electrons as compared to Cu_2_O, indicating less recombination of electrons and holes. Thus, Cu_2_O/Ru_*x*_O exhibited a 6-fold higher initial CO yield of 0.88 μmol g^–1^ h^–1^ than that of 0.16 μmol g^–1^ h^–1^ over pure Cu_2_O under a 150 W Xe irradiation. Furthermore, we also studied the different charge carrier kinetics among a family of g-C_3_N_4_ derived from various precursors by TAS and photoemission studies.^50^ The TAS results suggested that DCDA and thiourea-derived materials had a higher yield of deep-trapped carriers than that on urea-derived g-C_3_N_4_, thus leading a decreased photocatalytic activity. Besides, the steady-state emission studies revealed that the enhanced photocatalytic performance on urea-derived g-C_3_N_4_ was attributed to the increased driving force for electron transfer to the [Co(bpy)_n_]^2+^ cocatalyst. Hence, urea–g-C_3_N_4_ exhibited a superior CO yield of 460 μmol g^–1^ h^–1^ (300 nm < λ > 795 nm), which was 20 and 5 times higher than that on thiourea and DCDA-derived g-C_3_N_4_, respectively.

**Figure 8:**
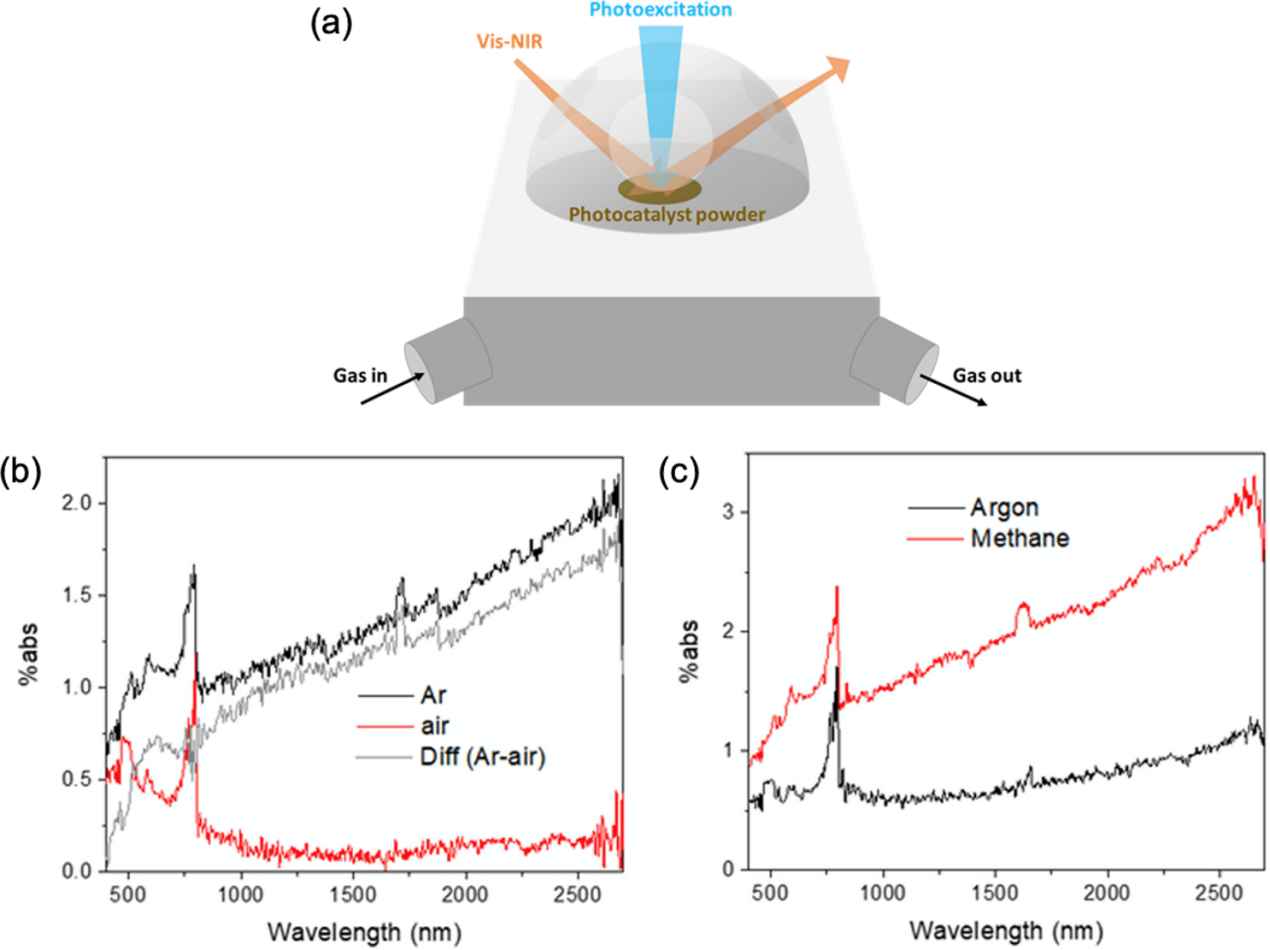
(a) Schematic diagram of the setup using an in situ vis–NIR spectroscopy system for methane photoactivation. Photoinduced adsorption spectra (PIA) on anatase TiO_2_ at 365 nm irradiation in the presence of (b) air and (c) 10% methane. Reproduced with permission from ref (52). Copyright 2021 American Chemical Society.

Lately, time-resolved photoluminescence (tr-PL) and TAS were employed together to study the photophysics of urea-derived carbon nitrides.^51^ The TAS spectra revealed that electrons on g-C_3_N_4_ were excited within 200 fs and then trapped in a pico time scale, followed by a power law decay that indicated a trapping–detrapping process. Additionally, with the assistance of the modeling analysis, it pointed out that the thermal equilibrium between the nonradiative trapped and the emissive states caused the faster decay of tr-PL compared to TAS decay. Further experimental and DFT results demonstrated the inverse correlation between the yield of deep-trapped long-live inactive electrons over g-C_3_N_4_ and the related H_2_ production. Most recently, we constructed an in situ vis–NIR spectroscopy system to elucidate the initial steps of methane photoactivation over anatase TiO_2_ (Figure 8a).^52^ From the in situ experimental study, under constant light irradiation, the intensity of photogenerated electrons on anatase TiO_2_ increased in the NIR region under methane atmosphere as compared to that under argon gas. Combined with the significantly decreased signal intensity of electrons under air condition (Figure 8b), it was strongly evidenced that methane was a hole scavenger in photocatalytic methane conversion (Figure 8c). In addition, the photoinduced absorption results revealed that 90 ± 6% of photogenerated electrons and 61 ± 9% of photogenerated holes were depleted by O_2_ (in dry air) and methane (10% in argon), respectively. Meanwhile, there was no obvious difference of the number of photoexcited electrons even if the ratio of O_2_ was reduced from 20% to 2%, which indicated that O_2_ was a much more easily activated component as compared to methane under light illumination.

**Figure 9:**
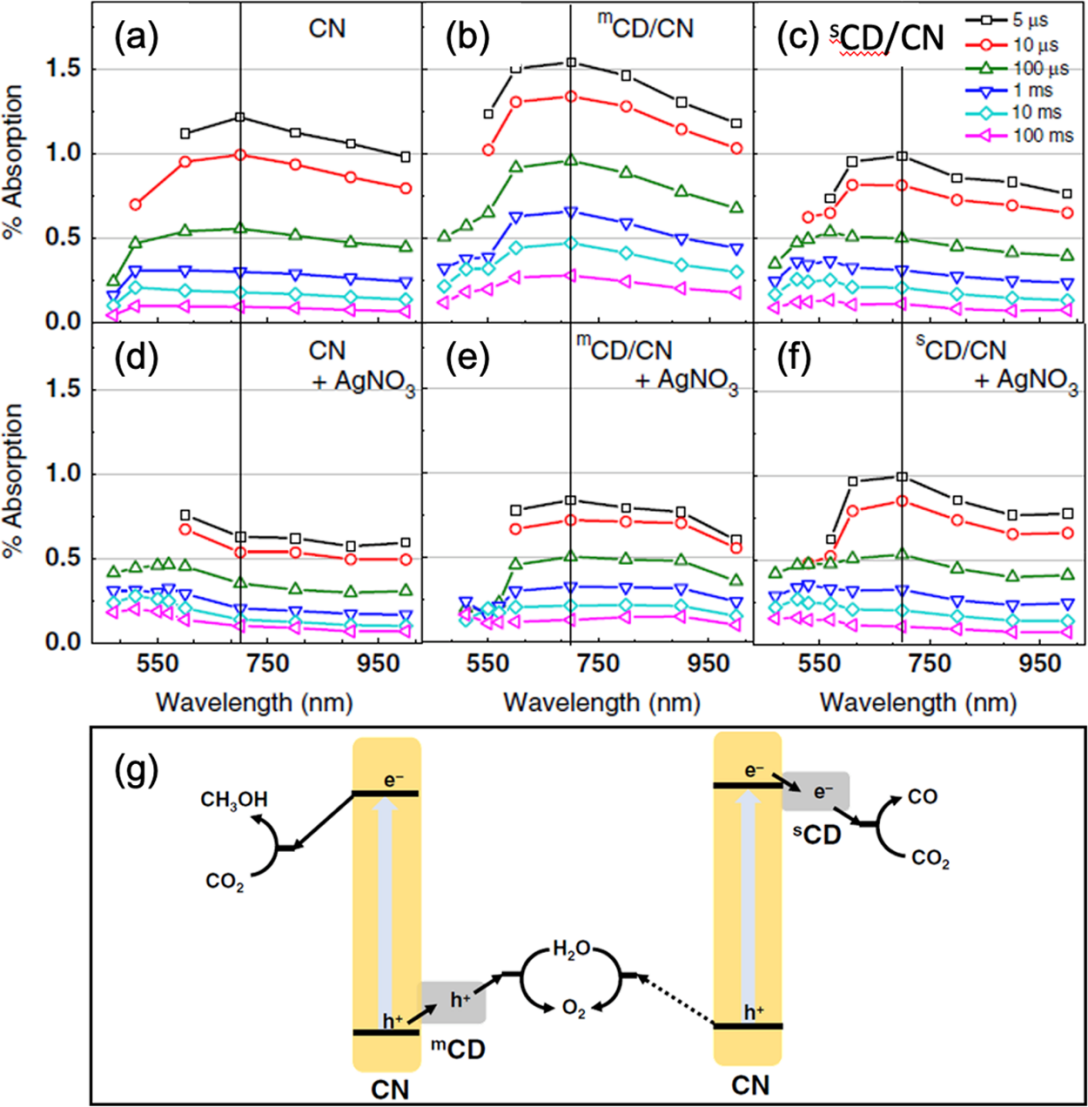
TAS spectra showing the electron signals of CN, ^m^CD/CN, and ^s^CD/CN (a–c) alone and (d–f) after addition of electron scavenger Ag^+^ and (g) reaction mechanism of ^m^CD and ^s^CD decorated CN for photocatalytic CO_2_ reduction. Reproduced with permission from ref (42). Copyright 2020 Springer Nature.

TAS was also used to study the charge separation and transformation process of carbon dot modified g-C_3_N_4_. Recently, a special hole-accepting carbon dot via the microwave method (^m^CD) was developed in the group.^42^ The main features on the TAS spectra indicated the electron signal observed at 700 nm in g-C_3_N_4_. As shown in Figure 9a–f, compared with pure g-C_3_N_4_ (CN), ^m^CD modified g-C_3_N_4_ (^m^CD/CN) displayed a stronger electron signal, indicating holes were effectively extracted from CN to ^m^CD. In contrast, carbon dots by the traditional ultrasonication method (^s^CD) decorated g-C_3_N_4_ (^s^CD/CN) gave a lower TAS intensity than pure g-C_3_N_4_, suggesting electron transfer from g-C_3_N_4_ to ^s^CD. The addition of Ag^+^, a strong electron scavenger, resulted in a decrease in the signal of CN and ^m^CD/CN, which was caused by the consumption of electrons on the surface of CN by Ag^+^. However, the spectra of ^s^CD/CN barely changed. All these suggested that electrons were transferred from CN to ^s^CD while holes were transferred from CN to ^m^CD (Figure 9g).

**Figure 10:**
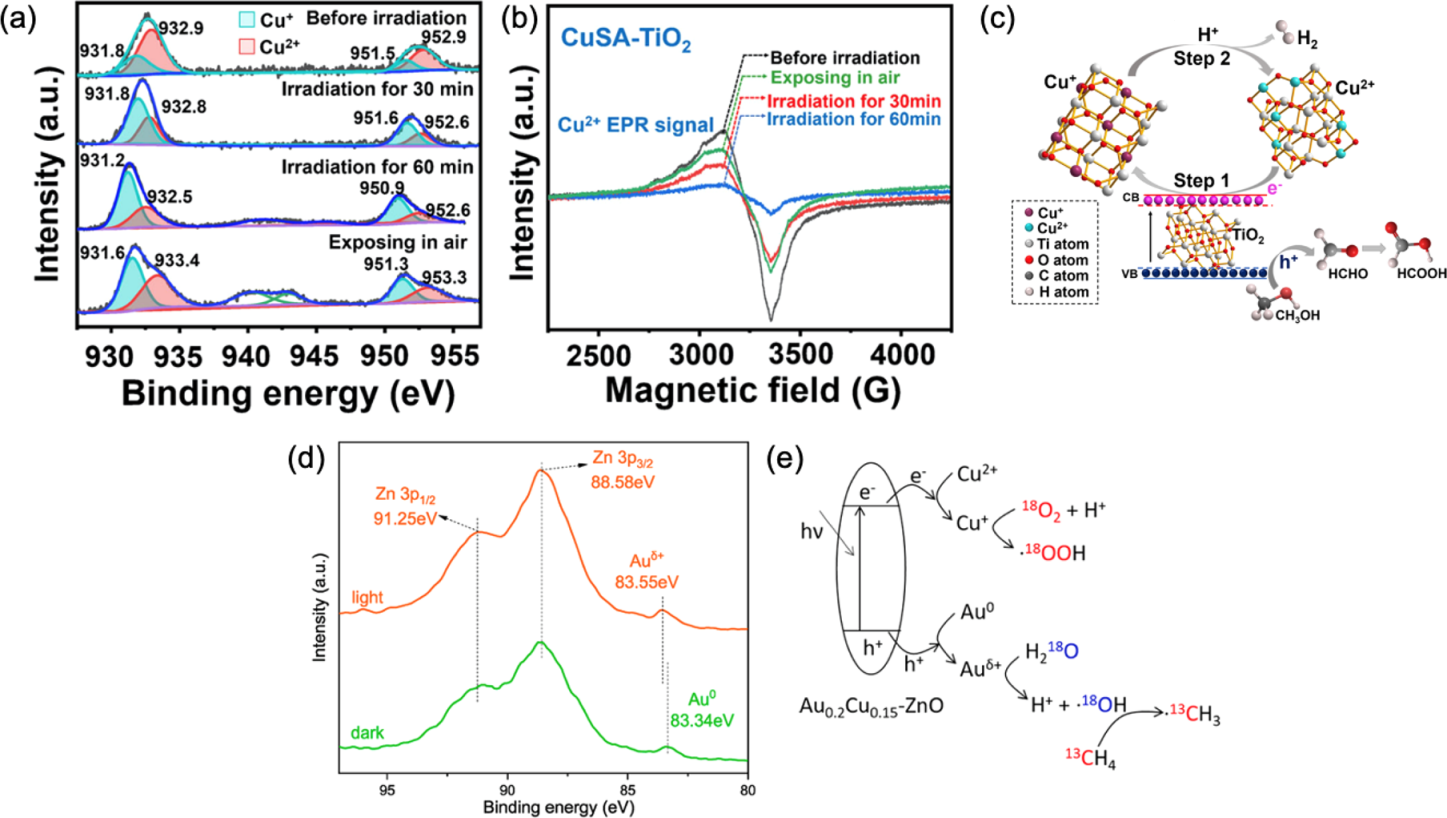
(a) In situ XPS spectra of Cu 2p and (b) in situ EPR spectra of CuSA-TiO_2_. (c) The reaction mechanism for photocatalytic H_2_ evolution on CuSA-TiO_2_. Reproduced with permission from ref (13). Copyright 2022 Springer Nature. (d) In situ XPS spectra of Au 4f on Au_0.2_Cu_0.15_-ZnO and (e) proposed reaction mechanism of photocatalytic methane conversion on Au_0.2_Cu_0.15_-ZnO. Reproduced with permission from ref (53). Copyright 2021 American Chemical Society.

The complementary in situ and operando experiments such as XAFS (X-ray absorption fine spectroscopy), XPS (X-ray photoelectron spectroscopy), EPR (electron paramagnetic resonance), etc. were also widely studied in the small molecule photoactivation process. Very recently, we employed the in situ XPS and EPR to monitor the real active sites on single-atom-copper-loaded TiO_2_ (CuSA-TiO_2_) for photocatalytic hydrogen production.^13^ As seen from the in situ XPS (Figure 10a), after 30 min light irradiation, the fraction of Cu^+^ in the sample increased dramatically from 29.42% to 61.68% and kept 62–66% after that. Then, the in situ EPR also confirmed the valence states of Cu in the CuSA-TiO_2_ (Figure 10b), where the CuSA-TiO_2_ exhibited a strong signal of Cu^2+^ before light irradiation. Nevertheless, the Cu^2+^ EPR signal was dramatically decreased after 60 min irradiation and then recovered after exposure to the air. All these results provide strong evidence that Cu^2+^ was the electron acceptor and then Cu^+^ was active sites to reduce proton for H_2_ production, while the photoinduced holes could oxidize methanol to produce the value-added chemicals including formaldehyde and formic acid, as shown in Figure 10c. Similarly, we also employed the in situ XPS to reveal the valence state of Au on ZnO for photocatalytic methane oxidation to C1 oxygenates.^53^ It could be seen that the XPS peak of Au_4f_ had a significant shift to a high binding energy under light irradiation (Figure 10d), which indicated that Au served as a hole acceptor. As a result, a reaction mechanism for photocatalytic methane oxidation was proposed as shown in Figure 10e, where Au cocatalysts accepted holes to form Au^δ+^, which further activated H_2_O to produce ^•^OH and H^+^.

Besides, the in situ XAFS is also a promising way to monitor the change of catalytic active sites under real reaction conditions.^54^ Very recently, the extend X-ray absorption fine structure (EXAFS) was employed in our group to prove the existence of a single Pd atom on Pd-loaded defective In_2_O_3_ with oxygen vacancies.^55^ Additionally, we also used the EXAFS technique to confirm the existence of the iron in the form of small clusters or even a single atom on the Fe-loaded TiO_2_ photocatalyst.^56^

## 3 Conclusions and Perspective

Photocatalytic conversion of small molecules into valued-added chemicals has been critically viewed, mainly taking research results achieved in our group as examples to indicate an efficient, environmentally friendly, and energy-saving strategy to mitigate the current energy and environment issues. The key factors in photoinduced small molecule conversion are to overcome the reaction energy barrier of small molecule activation and promote their adsorption and product desorption. Despite substantial effort in past decades, the conversion efficiency and selectivity to valuable chemicals by photocatalysis are still relatively low and cannot meet the requirements for the large-scale production. Thus, it is still at the infancy stage with a long way to go.

In this Account, we enumerate several strategies used in our group for enhanced photoactivity involving (i) light harvesting by bandgap engineering such as defects and elements doping, (ii) charge separation enhancement by cocatalysts loading and junction construction, (iii) reactants adsorption or product desorption together with charge separation by morphology engineering including facets and nanostructures, and (iv) fundamental understanding by in situ and operando investigations, which have been validated by us over the past two decades. To further facilitate these research areas, the future key challenges in the photocatalytic small molecule conversion processes are discussed below based on our understandings:

(i) Photocatalytic H_2_O splitting. Photocatalytic H_2_ production from water provides an attractive pathway to achieve green H_2_ production. Until now, much progress has been made in search of effective photocatalysts for H_2_O splitting. However, the photocatalytic efficiency under real sunlight is still relatively low (∼1%).^5^ Thus, it is urgent to design effective visible-light response photocatalysts. Besides, there are very few studies about the design of the monolith reactor for photocatalytic H_2_O splitting. Considering the realization of large-scale H_2_ production in the future, an effective and inexpensive photocatalytic reactor, together with a H_2_ and O_2_ gas separator are expected. Furthermore, in most cases, the sacrificial agents were used to improve the H_2_ yield in the photocatalytic H_2_ production system, whereas the real proton source and reaction pathway are still unclear. As a result, the isotope labeling experiments and in situ and/or operando techniques should be applied for mechanistic understanding in order to improve the solar to H_2_ conversion efficiency of above 10%, which is widely regarded as a threshold for the application of this technology.
(ii) Photocatalytic CO_2_ reduction. Although massive progress has been achieved in photocatalytic CO_2_ reduction, the low conversion rate of CO_2_ and selectivity toward valuable products are the main challenges faced in photocatalytic CO_2_ reduction. More attention should be paid to the surface reaction, for instance, reactant adsorption and activation, the formation and transformation of intermediates, and product desorption, as most of the current studies paid too much attention to the light harvesting and charge separation steps in photocatalysis. The reaction pathway and mechanism study can definitely promote the development of photocatalytic carbon dioxide reduction, which should be comprehensively investigated by combination of experiments and theoretical modeling.
(iii) Photocatalytic N_2_ reduction. To date, compared with other photocatalytic processes, N_2_ photoreduction suffers from serious challenges. First, the yield of NH_3_ is currently too moderate, in the magnitude of a micromole or even below, close to the natural NH_3_ concentration in air or in river water.^57^ Thus, more effort should be paid to new photocatalyst designs such as dual metal photocatalyst and polymer/oxide junction to enhance N_2_ adsorption and activation. Second, one robust and reliable detection procedure of NH_3_ yield should be applied to avoid the interference of environmental impurities or detection agents. Besides, the current study for N_2_ photoreduction occurs in lab-based batch reactors, where the NH_3_ yield is usually restricted by the mass transfer among N_2_, H_2_O, and solid catalysts due to the extremely low solubility of N_2_ in water. Thus, a novel flow reactor integrating gas reactants and solid photocatalysts is expected, which might show great potential for future large-scale application. Last, the reaction pathways for N_2_ photoreduction over semiconductors is currently unclear and less investigated. The in situ and/or operando techniques, together with computational modellings should be explored in order to give clear guidance for future effective photocatalysts synthesis and reaction system design.
(iv) Photocatalytic CH_4_ conversion. Besides photocatalytic activation of inert inorganic molecules discussed above, organic molecule conversion is the other key research subarea in photocatalysis. Methane, the smallest organic molecules, can be converted to methanol, ethane, ethylene, ethanol, and other oxygenates by photocatalysis. Due to the inert nature of methane and the high reactivity of products, the selectivity toward desired products is relatively moderate, although the feasibility has been proved. Therefore, improving the selectivity by controlling overoxidation or coking should be the main task in the study of photocatalytic methane conversion. Furthermore, oxygen or air is a preferable oxidant for partial oxidation of methane, instead of peroxide, carbon monoxide, or nitrogen oxide used in many studies. In addition, only some UV-responsive photocatalysts were used in methane conversion. Photocatalysts with higher visible light efficiency should be developed. Currently, C1 and C2 products have been reported as major products by photocatalytic methane conversion. More valuable products, such as oxygenates and hydrocarbons with longer carbon chains, are encouraged to be selectively produced. The complex radical reactions and intermediates formed during the methane conversion process should be studied using advanced spectroscopies and microscopies. In situ and operando characterizations should be applied to understand the true reaction sites and key intermediates formed at the catalysts surface. These will greatly guide the research directions in photocatalytic methane conversion.
(v) Photocatalytic alcohol and benzene oxidation. The real application of selective photocatalytic oxidation of organics is our last, but not least, subject of interest. It presents some difficulties compared with the degradation of organic pollutants. The primary issues in this subject are the control of the reactive oxygen species (ROS) generation and the understanding of reaction mechanisms. Despite the relatively fast progress in the development of catalysts for photocatalytic oxidation of organic substances in the past few decades, there are still challenges to be overcome in this field. For example, the operando observation of the reaction intermediates, related to the catalytic reaction mechanism is highly desirable. Combining time-resolved IR and Raman spectroscopy with mass spectrometry, one can observe species adsorbed on the photocatalyst surface, species in the gas phase, and subtle surface changes of catalysts under “true” reaction conditions, leading to clarification of the photooxidation mechanisms.^58^ Based on these experimental results, theoretical calculation is the next essential step to confirm the reaction mechanism, which can also predict effective strategies for catalyst optimization.

Overall, the core of these photochemical processes is the discovery of effective, stable, and low cost photocatalysts. State-of-the-art methods for modifying the electronic and chemical structures of photocatalysts should be further developed and employed. Specifically, single-atom catalysts (SACs), which can potentially bring exclusively high activity and selectivity in photocatalytic organic synthesis, are still scarcely employed in this field. Very recently, we have successfully developed several single-atom photocatalysts for the methane oxidation to methanol and water reforming of methanol for hydrogen evolution, both achieving very high photocatalytic activity.^13,56^ It is therefore of paramount importance to develop new strategies to controllably synthesize SACs with high metal content in an appropriate circumstance. On the other hand, SACs can provide new opportunities to capture reaction intermediates at the atomic scale and further monitor the dynamic behaviors of both the geometric structure and electronic state of the active sites. Furthermore, the operando study on SACs can achieve the atomic-level knowledge of these active sites, leading to deep and comprehensive understanding of the reaction mechanisms. Furthermore, the big data, together with artificial intelligence and machine learning are becoming a very promising research field for novel photocatalyst development, which can effectively optimize experimental process, save experimental time, reduce raw materials consumption, and enhance the experimental safety. Therefore, the combination of the data-related diverse technologies should be paid particular attention for photocatalysis in future studies.
